# *In Silico* Study of Anti-Insomnia Mechanism for Suanzaoren Prescription

**DOI:** 10.3389/fphar.2019.00925

**Published:** 2019-08-22

**Authors:** Jian Gao, Qiming Wang, Yuwei Huang, Kailin Tang, Xue Yang, Zhiwei Cao

**Affiliations:** ^1^Shanghai 10th People’s Hospital, School of Life Sciences and Technology, Tongji University, Shanghai, China; ^2^Department of Traditional Chinese Medicine, Yangpu Hospital, School of Medicine, TongJi University, Shanghai, China

**Keywords:** network pharmacology, suanzaoren prescription, insomnia, bioinformatics, systematic analysis

## Abstract

Insomnia is a common and widespread sleeping disorder caused by various risk factors. Though beneficial, conventional treatments of insomnia have significant limitations. As an alternative treatment, Chinese herbal formula Suanzaoren prescription (SZRP), composed of Suanzaoren [seeds of *Ziziphus jujuba* var. *spinosa* (Bunge) Hu ex H.F.Chow] and four additional herbs, has been reported with significant anti-insomnia effects. Yet the anti-insomnia mechanism of the herb formulae remains unknown. In this study, we attempted to extrapolate the holistic anti-insomnia mechanism of SZRP through herbal targeting and network pharmacology. The results indicated that the ingredients of Suanzaoren can target multi-neurotransmitter receptors at synapse interface, which was reported to be associated with sedative and hypnotic effects, while the four additional herbs can hit multiple pathways downstream of membrane neurotransmitters. Furthermore, the four additional herbs showed highly cooperative targeting patterns in the paralleled and cross-talked pathways related to inflammatory regulation and endocrine system, which may contribute to the additional relief of insomnia caused by inflammation, anxiety, or endocrine disorder. The interesting complementary mechanism we found among the herbal groups of SZRP may provide an example to study Chinese herbal formula and offers clues to future design of anti-insomnia strategy.

## Introduction

Insomnia is a common sleep disorder in which people have difficulty falling asleep or staying asleep (Punnoose et al., 2012). It can sap quality of life and even cause damage to health. Short-term insomnia can cause daytime sleeping and low energy (Lande and Gragnani, 2010), while long-term insomnia may lead to serious problems including driving accident, anxiety, chronic pain, cardiovascular disease, and heart failure (Taylor et al., 2007). According to its etiology, insomnia can be classified as primary or secondary (National Heart, 2016). Primary insomnia is not directly associated with any other health conditions or problems, and its pathogenesis remains unclear. On the contrary, secondary insomnia is related to those obvious triggers, and it is generally caused by the following reasons: 1) emotional disorder including psychological stress, depression, and anxiety; 2) health conditions of chronic pain, such as arthritis, headache, and other inflammations; 3) hormone disorder including menstruation, menopause, and hyperglycemia (Pepin et al., 2014; Santoro et al., 2015); and 4) some medicines and substances.

Anti-insomnia treatment often includes lifestyle change, cognitive-behavioral therapy, and prescription medicines (Buysse, 2013). Nonpharmacologic treatments are usually recommended as the first-line treatment (Trauer et al., 2015), while pharmacologic treatments including benzodiazepines and benzodiazepine-like medications are applied as additional treatment if nonpharmacologic treatments fail (Asnis et al., 2015). Though pharmacologic treatments can be effective, patients may not tolerate their side effects such as rebound withdrawal effects, disruption in sleep architecture, grogginess, memory impairment, and undesired behaviors during sleep (Tiller et al., 2003; Wilt et al., 2016). In addition to those drugs mentioned above, antidepressants, antipsychotics, and antihistamines may also be applied for sedative effect. Yet they are usually not recommended in the absence of corresponding disease symptoms. Due to the present limited treatment, a substantial number of patients worldwide have started to seek their sleeping aids in herbal medicine as an alternative approach (Chen et al., 2009; Frass et al., 2012). It has been reported that Chinese herbal formulae “Xiao Yao Wan,” “liquorice, wheat, and jujube soup,” “Tian Wang Bu Xin Dan,” and “Suanzaoren prescription (SZRP)” are clinically effective against insomnia (Chen et al., 2011; Liu et al., 2015; Ko et al., 2016); among them, SZRP is the most widely used and well documented for refractory insomnia. It was first recorded in *Shennong Bencao Jing*, which is the earliest authoritative monograph on pharmacy in China (Gu, 2007). In addition to the clinical practice, high-quality randomized controlled trials have confirmed its efficacy and safety (Wang et al., 2010; Wang et al., 2013; Yuan et al., 2013). Moreover, a meta-analysis study covering 1,454 patients showed that both SZRP monotherapy and combinational treatment can improve sleep quality significantly with minimal side effects (Zhou et al., 2018).

SZRP contains the main herb seeds of *Ziziphus jujuba* var. *spinosa* (Bunge) Hu ex H.F.Chow (Suanzaoren) and four additional herbs: *Anemarrhena asphodeloides* Bunge (Zhimu), *Wolfiporia extensa* (Peck) Ginns (Fuling), *Ligusticum sinense* Oliv. (Chuanxiong), and *Glycyrrhiza uralensis* Fisch. (Zhigancao). According to the TCM theory and Chinese Pharmacopeia (China, 2015), Suanzaore is the *Emperor* (*Jun*), Zhimu and Fuling are the *Ministers* (*Chen*), Chuanxiong is the *Adjuvant* (*Zuo*), and Zhigancao is the *Courier* (*Shi*) (WHO, 2007). The functions of herb are usually believed to be multiple because of the vast chemical diversity. Previous literatures have reported that Suanzaoren is anti-insomnia (Shergis et al., 2017), Zhimu is laxative and anti-inflammatory (Park et al., 2018; Ji et al., 2019; Li et al., 2019), Fuling is diuretic (Zhao et al., 2012), and Chuanxiong is anti-migraine (Shan et al., 2018). However, current studies about the pharmacological mechanism of SZRP are mainly focused on the main herb Suanzaoren. Photochemical analysis indicated that Suanzaoren contains flavonoids, saponins, and triterpenes. Jujuboside A, sanjoinine A, and flavonoids in Suanzaoren were reported to have sedative and hypnotic effects. Further study showed that jujuboside A affected GABAergic and serotonergic system in rat through glutamate-mediated excitatory signal pathway (Cao et al., 2010). The hydrolysis product of jujuboside A, jujubogenin, was predicted to have high potential of blood–brain barrier penetration ability (Chen et al., 2008). In addition, sanjoinine A was found to be able to prolong sleeping time through increasing chloride influx and GABA synthesis (Zhang et al., 2003). In addition, the flavonoid 6-hydroxyflavone in Suanzaoren showed GABA agonistic action by binding to GABAA receptors (Ren et al., 2010). These aforementioned studies provide a glimpse of the partial mechanism of anti-insomnia effects of Suanzaoren herb. The holistic mechanism of SZRP formulae remains unclear. What is the mechanism of additional herbs in treating insomnia? How can they help to improve the therapeutic effects of the main herb? No systematic studies have been published so far. In this work, first, we conducted a global analysis of all known targets of herbal ingredients for five herbs of SZRP to elucidate the overall anti-insomnia mechanisms of SZRP, and then we analyzed the targeting network patterns herb by herb for better understanding of their potential roles in TCM formulae.

## Methods

### Dataset

The information of herbal ingredients and targets was collected from online TCM databases Traditional Chinese Medicine Systems Pharmacology (Ru et al., 2014), Herb Ingredients’ Targets (HIT) (Ye et al., 2011), and Natural Product Activity & Species Source Database (NPASS) (Zeng et al., 2018). The following key words were used to search in these databases: *Ziziphus jujuba* var. *spinosa* (Bunge) Hu ex H.F.Chow OR Suanzaoren; *Anemarrhena asphodeloides* Bunge OR Zhimu; *Wolfiporia extensa* (Peck) Ginns OR Fuling; *Ligusticum sinense* Oliv. OR Chuanxiong; and *Glycyrrhiza uralensis* Fisch. OR Zhigancao. Therapeutic targets were collected from Therapeutic Targets Database (TTD) (Li et al., 2018). The validated information of plants was collected from Kew Royal Botanic Garden (https://mpns.science.kew.org/mpns-portal/) and The Plant List (http://www.theplantlist.org).

### Functional Annotation and Enrichment Analysis

Statistical analysis of KEGG function enrichment of the target profile was performed by Metascape (Tripathi et al., 2015) (http://metascape.org). The pathways significantly enriched were selected (*p*-value < 0.01). Then only terms with both −Log (*p*-value) > 5 and more than 5% targets falling into the category were retained. The retained terms were mapped into bubble graph by “pyplot” of matplotlib. Finally, bubble graphs of each herb were combined and labeled with Adobe Photoshop software (Adobe, San Jose, California).

### Network Construction for SZRP

To better elaborate the holistic mechanism of SZRP, three subnetworks were compiled by following the procedures: 1) All targets of SZRP were submitted to an online tool KEGG Search Pathway (https://www.genome.jp/kegg/tool/map_pathway1.html). 2) All result maps of KEGG were downloaded and integrated. 3) Maps related to “nervous,” “immune,” or “endocrine” were reserved. 4) For each label of “nervous,” “immune,” and “endocrine,” multiple pathways were integrated and overlapped according to cross-talk targets in these maps. Detailed information such as targets names and integrated pathways are shown in **Table S3**. 5) Subnetworks were drawn with Adobe Illustrator software, where intermediate genes were hidden for better display.

## Results

### Overall Ingredients and Targets of SZRP

**Figure 1:**
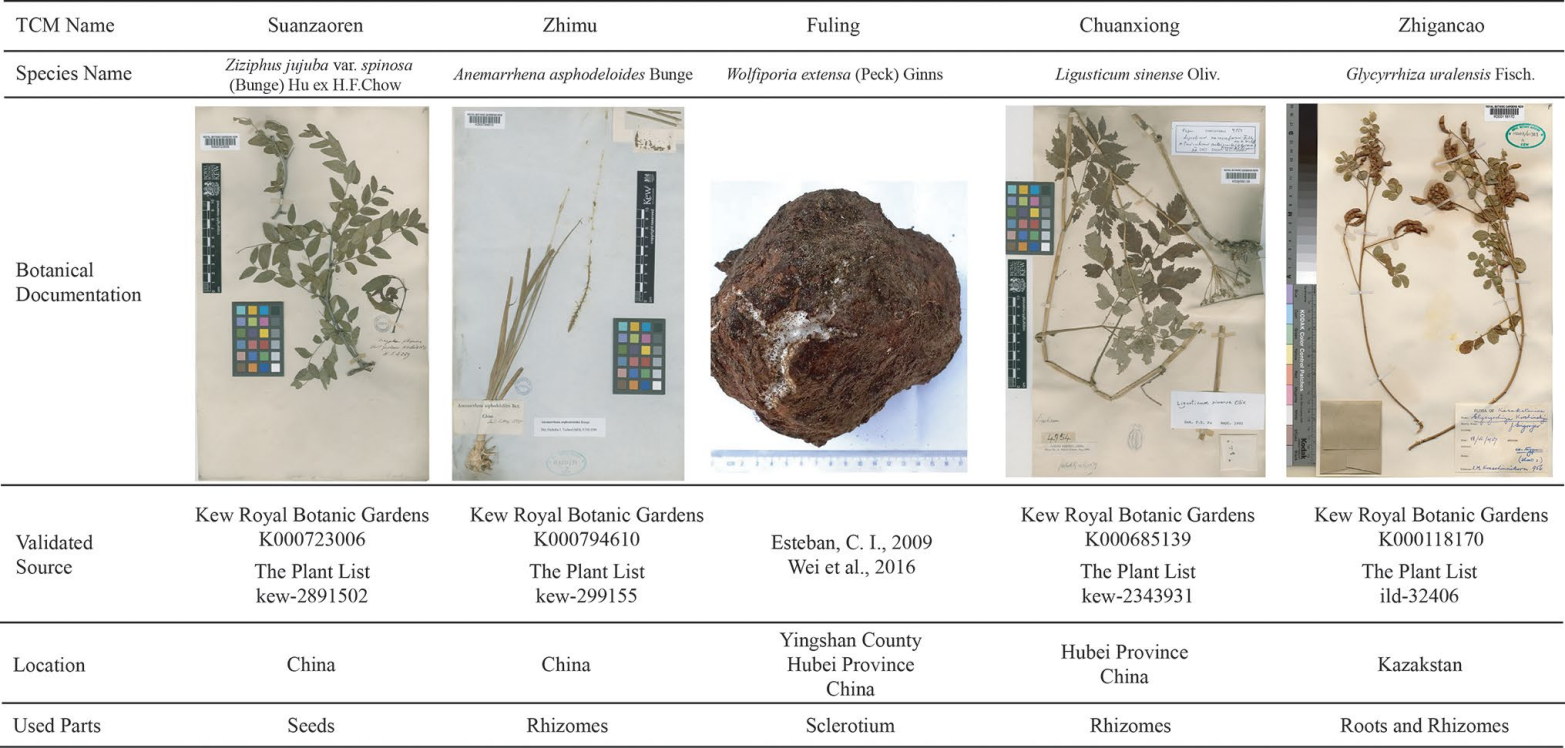
The validated information of five herbs in SZRP, including botanical documentation (voucher specimen deposited in herbarium), location (the collection location of voucher specimen), and used part (used part of medicinal species).

**Figure 2:**
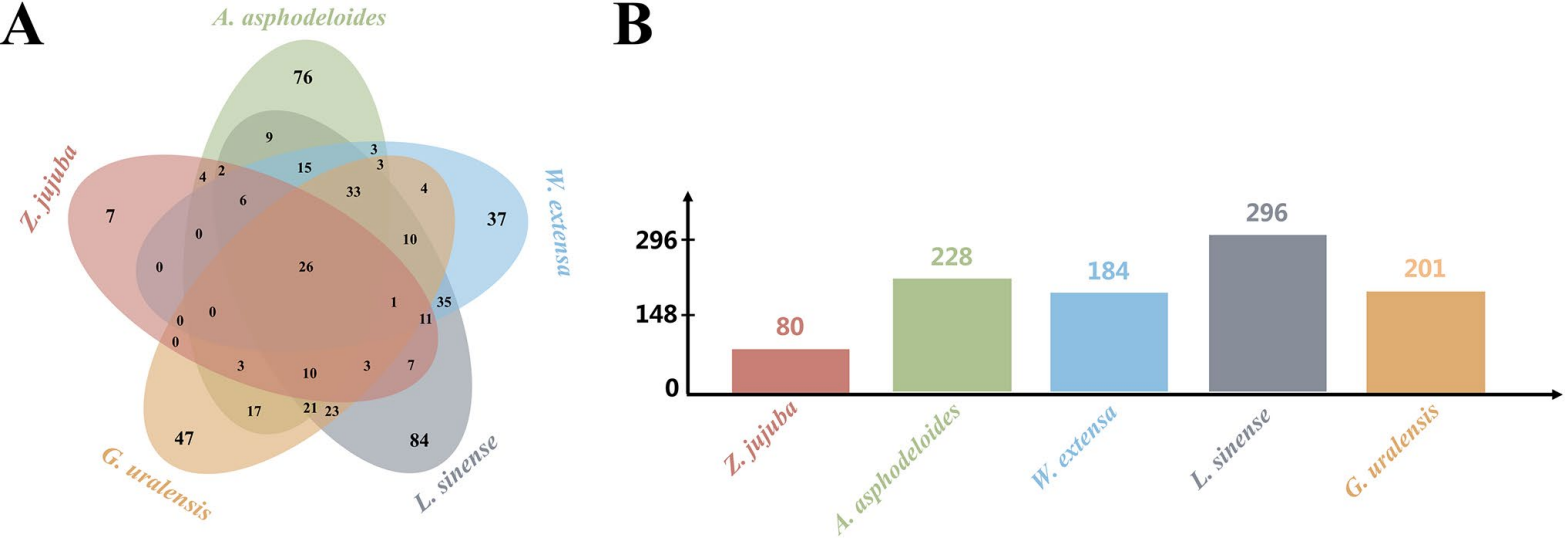
The target distribution of five herbs. **(A)** Unique and overlapping target of five herbs. **(B)** The number of targets for different herbs.

To understand the potential anti-insomnia mechanism of SZRP holistically, known targets for herbal ingredient in the formula were collected and analyzed. Five medicinal species were validated by botanical documentation (**Figure 1**). *Z. jujube*, *A. asphodeloides*, *L. sinense*, and *G. uralensis* were validated by Kew Database and TPL. *W. extensa* was reported as an edible fungus (Esteban, 2009; Wei et al., 2016). Through database searching, 497 unique targets were collected for five herbs in SZRP (**Table 1**); 24% of proteins (119) were reported as therapeutic targets in TTD (Li et al., 2018). Three known therapeutic targets of anti-insomnia drugs were covered by SZRP targets including gamma-aminobutyric acid type A receptor (GABAR), 5-hydroxytryptamine receptor (HTR), and histamine receptor (HRH). All information of targets is shown in **Table S1**. The distribution of targets of five herbs is shown in **Figure 2**. Eighty known targets were retrieved for Suanzaoren, among which 26 were shared by all five herbs. In terms of target abundance, *A. asphodeloides* retrieved the most targets, while *L. sinense* had the most abundant unique targets.

**Table 1:** Number of ingredients and known targets for each herb in SZRP.

Species name	TCM name	Ingredients	Targets
*Ziziphus jujuba* var. *spinosa* (Bunge) Hu ex H.F.Chow	Suanzaoren	35	80
*Anemarrhena asphodeloides* Bunge	Zhimu	82	228
*Wolfiporia extensa* (Peck) Ginns	Fuling	58	184
*Ligusticum sinense* Oliv.	Chuanxiong	292	296
*Glycyrrhiza uralensis* Fisch.	Zhigancao	272	201
**Total (SZRP)**		**711**	**497**

### Functional Analysis of Separated SZRP

**Figure 3:**
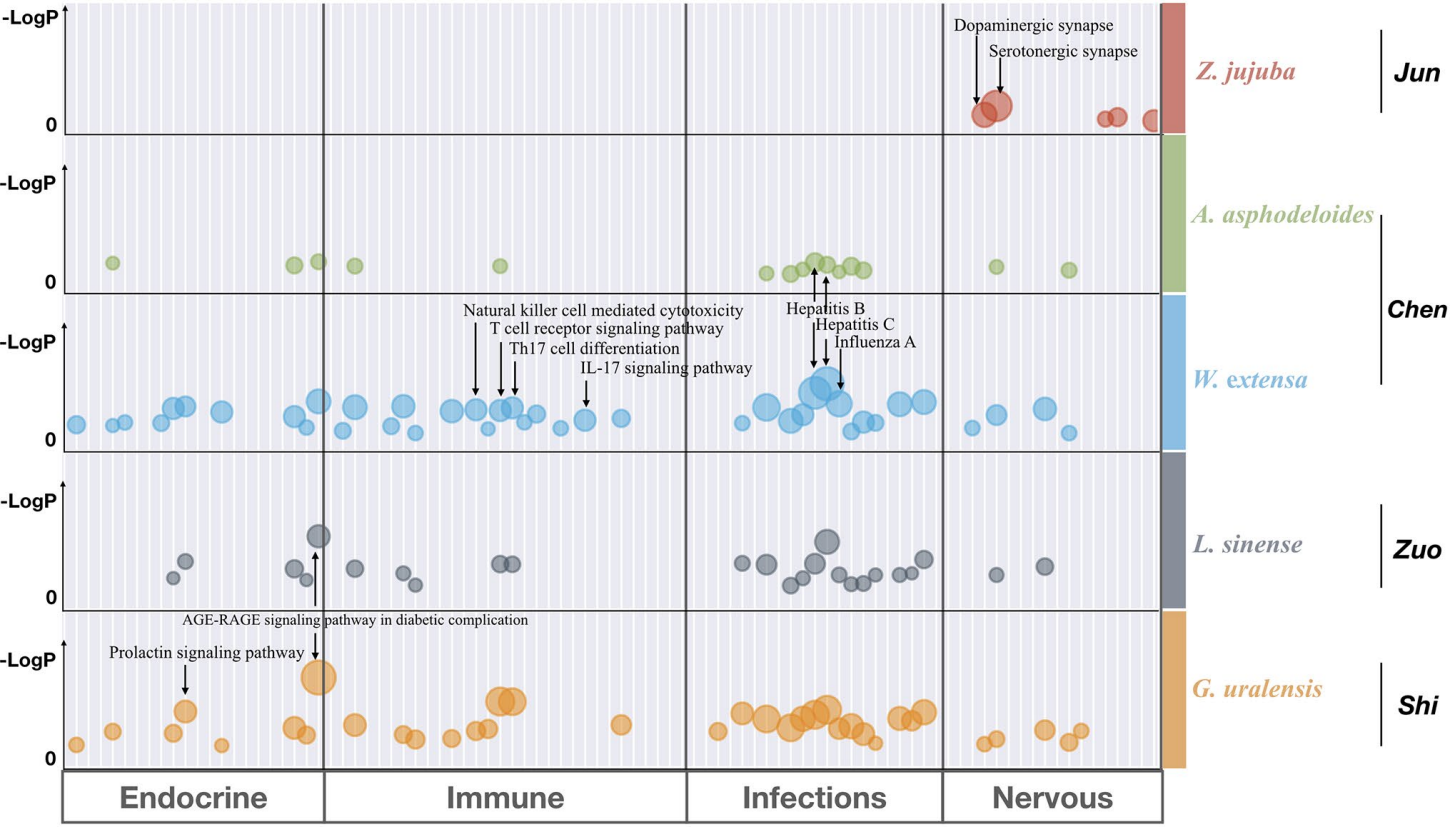
Bubble graph for significantly enriched pathway terms from KEGG by Metascape. Each bubble represents one KEGG pathway term. The size of the bubble correlates with the relative ratio of targets hitting each pathway over the total targets, with larger bubbles indicating the more enriched terms. The LogP shows the statistical significance of *p*-values, with larger and higher numbers indicating the more significant ones. And only those functional terms were retained with both −Log(*p*-value) > 5 and enrichment ratio > 5%. The detailed statistical data are shown in **Table S2**.

To investigate the functional relationship among the five individual herbs of SZRP, targets of each herb were mapped to KEGG for functional enrichment analysis. The top 5 functional pathways for each herb in SZRP are listed in **Table 2**. It can be seen that apart from the main herb, the top enriched pathway profiles were highly similar for the four additional herbs. In **Table S2**, the overall pathway enrichment of total formulae is shown with their targets combined. Aside from cellular processes, metabolism, and signal transduction terms, organismal systems of nervous, endocrine, immune infections (NEIs) were significantly enriched. As our aim is to interpolate the potential anti-insomnia mechanism, we tentatively focused on organismal systems of NEIs, which are known to closely relate with insomnia pathology (Santoro et al., 2015; Xiang et al., 2019). To display the functional similarity and difference among herbal groups of SZRP, significant pathway terms of KEGG were mapped into a bubble graph in **Figure 3**.

**Table 2:** Top five pathways enriched by targets of each herb in SZRP.

Species name	Herbal group	Pathway name	KEGG ID	Ratio (%)	−Log(*p*-value)
*Ziziphus jujuba* var. *spinosa* (Bunge) Hu ex H.F.Chow	*Emperor* (*Jun*)	Serotonergic synapse	hsa04726	12.5	11.09
		Dopaminergic synapse	hsa04728	10	7.7
		Amphetamine addiction	hsa05031	7.5	6.82
		Adrenergic signaling in cardiomyocytes	hsa04261	8.75	6.09
		Cocaine addiction	hsa05030	6.25	6.08
*Anemarrhena asphodeloides* Bunge	*Minister* (*Chen*)	Fluid shear stress and atherosclerosis	hsa05418	8.81	16.73
		AGE-RAGE signaling pathway	hsa04933	6.17	11.88
		Influenza A	hsa05164	7.49	11.67
		Ovarian steroidogenesis	hsa04913	4.85	11.65
		Adipocytokine signaling pathway	hsa04920	5.29	11.36
*Wolfiporia extensa* (Peck) Ginns	*Minister* (*Chen*)	Hepatitis B	hsa05161	13.66	25.55
		Influenza A	hsa05164	13.11	22.1
		AGE-RAGE signaling pathway	hsa04933	9.84	18.85
		Chagas disease (American trypanosomiasis)	hsa05142	9.84	18.6
		Hepatitis C	hsa05160	10.38	17.93
*Ligusticum sinense* Oliv.	*Adjuvant* (*Zuo*)	AGE-RAGE signaling pathway	hsa04933	9.18	27.63
		Hepatitis B	hsa05161	9.86	25.51
		Fluid shear stress and atherosclerosis	hsa05418	8.84	21.77
		Chagas disease (American trypanosomiasis)	hsa05142	7.14	18.79
		Prolactin signaling pathway	hsa04917	6.12	18.05
*Glycyrrhiza uralensis* Fisch.	*Courier* (*Shi*)	AGE-RAGE signaling pathway	hsa04933	14	33.98
		Fluid shear stress and atherosclerosis	hsa05418	15	32.19
		Th17 cell differentiation	hsa04659	11.5	24.88
		IL-17 signaling pathway	hsa04657	11	24.81
		Hepatitis B	hsa05161	11.5	21.73

The big and higher bubbles in **Figure 3** represent those highly significantly enriched pathway terms. It can be seen that the main herb of SZRP significantly regulates the function of nervous system, such as dopaminergic and serotonergic synapse pathways, suggesting that the *Emperor* (*Jun*) herb may enable hypnotic effects through modulating excitement in nervous system. In contrast, four additional herbs concentrated more significantly on the endocrine system, immunology system, and infections, offering complementary effects against insomnia. Specifically, the *Minister* (*Chen*) herb significantly targets virus infection pathways and immune cell pathways, while the *Adjuvant* (*Zuo*) and* Courier* (*Shi*) herbs affected signaling regulation of oxidative stress (AGE–RAGE) and hormone secretion (Prolactin).

### Subnetwork of SZRP on NEI System

**Figure 4:**
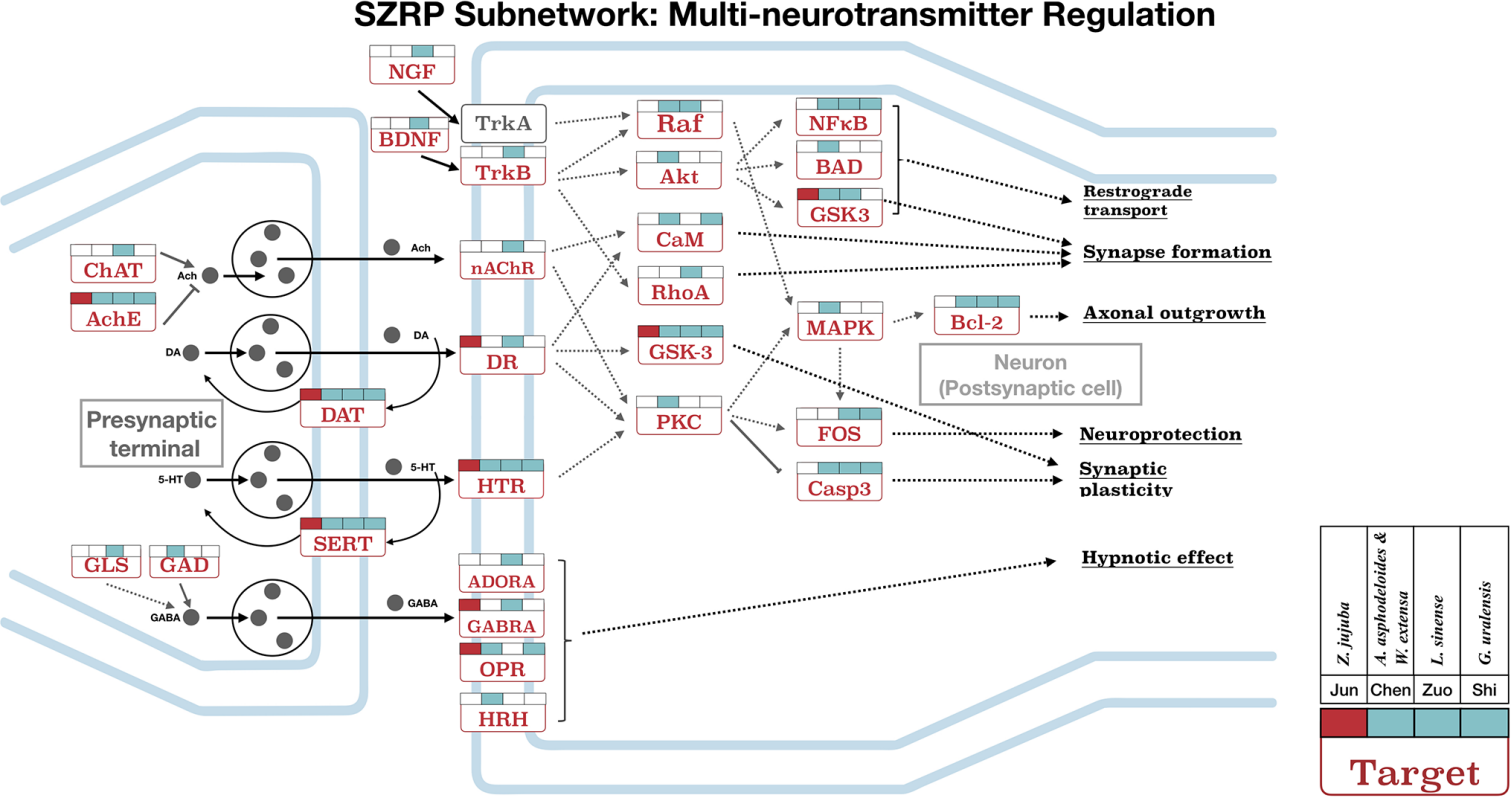
The targeting subnetwork showing multi-neurotransmitter regulation at both pre-synapse and post-synapse for SZRP. Target names are shown in rectangles, and their full gene names can be found in **Table S3**. The gray dots illustrate neurotransmitter/hormone. The colored flags above each target show the targeting pattern from four herbal groups of SZRP, where the *Emperor*’s (*Jun*) targets are labeled in red. The solid arrows in the figure mean directed actions, and dashed arrows represent intermediate genes abbreviated for better illustration.

**Figure 5:**
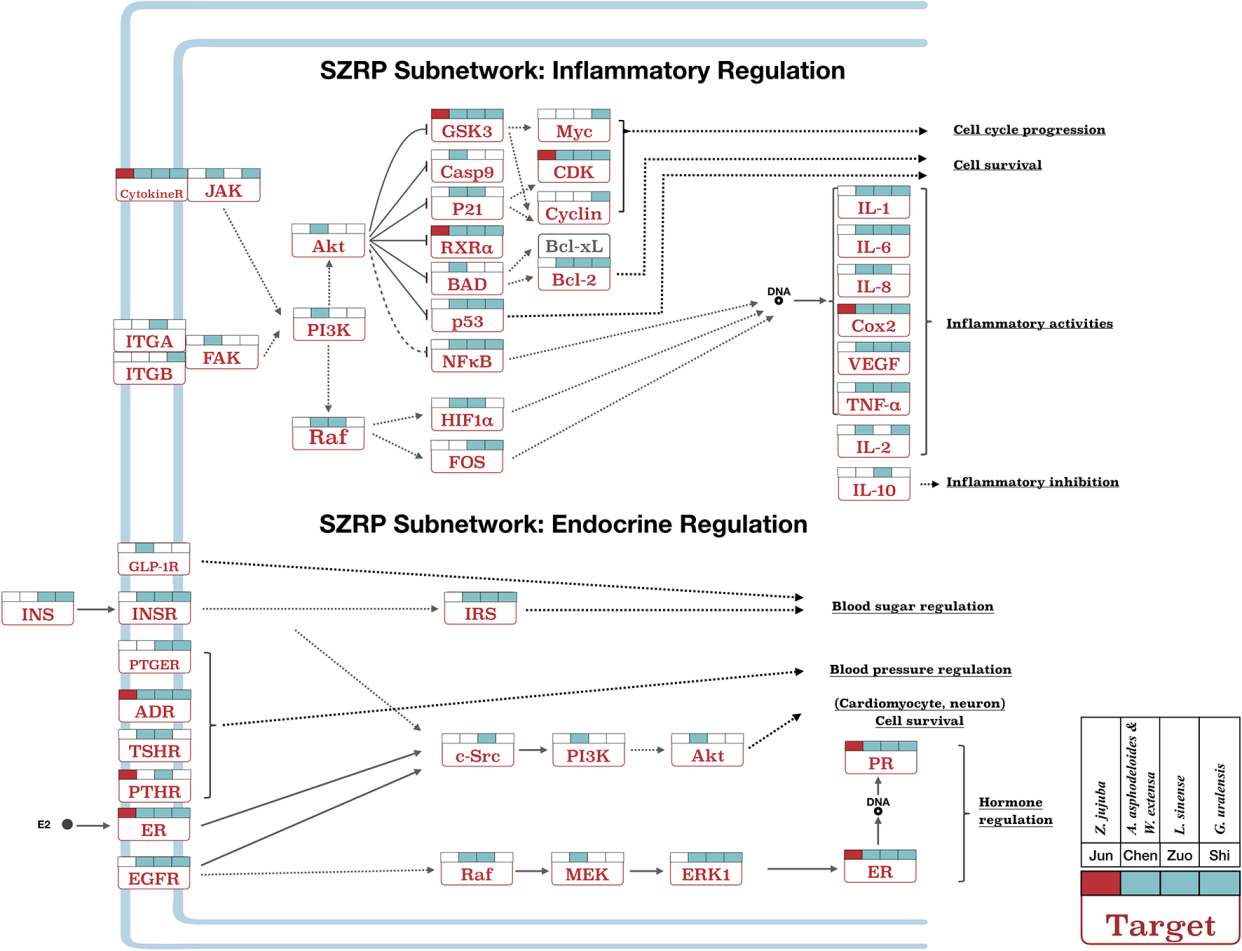
The pathway of inflammatory and endocrine regulation effect in SZRP. Target names are shown in rectangles, and their full gene names can be found in **Table S3**. All labels are same as **Figure 4**.

For further investigation, subnetworks were constructed based on enriched pathways for multi-neurotransmitter regulation, inflammatory regulation, and endocrine regulation (**Figures 4** and **5**). Each target in the pathways was labeled according to the different targeting patterns of the four herbal groups.

**Figure 4** shows the targeting patterns of SZRP herbs in a multi-neurotransmitter regulation network. These targets can be roughly divided into three classes: 1) interacted directly with multi-neurotransmitter receptors and downstream pathways; 2) related to the synthesis, secretion, and recycling of neurotransmitters; and 3) related to the sedative or hypnotic effects, such as opioid receptor (OPR) and adenosine receptor (ADORA). Of interest is that all herbal groups can target transporter proteins of dopamine and serotonin in the pre-synaptic membrane. When being examined from the perspective of herbal groups, the *Emperor* (*Jun*) herb significantly targeted on interfacial neurotransmitter receptors (**Figure 4**). The four additional herbs not only targeted those receptors but also targeted those in downstream, such as Raf, Akt, PKC, and CaM. Specifically speaking, the *Courier* (*Shi*) herb mainly regulated major downstream targets of multi-neurotransmitter regulation pathway. The synapse formation, axonal outgrowth, synaptic plasticity, and neuroprotection medicated by targets nAChR, DR, and HTR may be beneficial to maintain circadian rhythm; in addition, targets of SZRP such as GABRA, ADORA, and HRH have been reported to participate in sleep induction (Huang et al., 2014; Wisden et al., 2017).

Besides, SZRP can regulate immune-related inflammatory pathways and their upstream targets (**Figure 5**). The *Emperor* (*Jun*) herb mainly influenced COX2, while the four additional herbs took part in the regulation of inflammatory factors such as IL-1, IL-2, IL-6, IL-8, and IL-10. And the *Courier* (*Shi*) herb covered the largest area of inflammation pathways. Two compounds from the *Adjuvant* (*Zuo*) herb *L. sinense* have been reported to inhibit inflammation through downregulating IL-1 and IL-8 (Diodovich et al., 2003; Medeiros et al., 2007) and upregulating IL-10 (Sarkar et al., 2006). Inflammation and chronic pain are widely reported with insomnia (Cheatle et al., 2016), while inhibition of inflammation and chronic pain was shown to improve sleep quality (Trenkwalder et al., 2017; Wells et al., 2017).

Endocrine disorder associated with premenstrual and menopausal syndromes in women shows outstanding effects on secondary insomnia (Santoro et al., 2015). **Figure 5** shows the targeting patterns of SZRP herbs in endocrine regulation pathways. These targets were related to the regulation of blood sugar, blood pressure, and hormone system. All herbal groups mainly exerted a wide range of hormonal regulating effect by targeting the key receptors such as estrogen receptor (ER), progesterone receptor (PR), and adrenergic receptor (ADR). The whole formula played holistic and complementary roles in blood pressure and hormone regulation, while other herbal groups of *Minister* (*Chen*), *Adjuvant* (*Zuo*), and *Courier* (*Shi*) showed unique effects on blood sugar regulation by targeting insulin (INS), insulin receptor (INSR), and glucagon-like peptide 1 receptor (GLP-1R). Menstruation- and menopause-related insomnia is often caused by endocrine disorder (Santoro et al., 2015); moreover, hyperglycemia and hypertension are the high-risk factors of insomnia.

To understand the mechanistic relationship within TCM formulae, SZRP was disassembled into individual herbal groups. The *Emperor* (*Jun*) herb was found to mainly target receptors in synapse membrane, which might relieve the primary insomnia. The *Minister* (*Chen*) herbs can target not only the nervous system helping the *Emperor* (*Jun*) herb but also inflammation and endocrine pathways, providing potential assistance to secondary insomnia. Zhimu has been reported to act on HRH, an effective target for sedative effect (Krystal et al., 2013). Among the herbs, only the Adjuvant (Zuo) herb can upregulate IL-10, the well-known anti-inflammatory cytokine (Ouyang et al., 2011; Iyer and Cheng, 2012). It is noted that no unique function has been found for the *Courier* (*Shi*) herb in the network we constructed. Its function may be relates to the pharmacokinetics. Previous study showed that the *Courier* (*Shi*) herb was able to facilitate the adsorption of Suanzaoren (Shen et al., 2012; Bi et al., 2014). These results agree with the TCM theory that *Jun*, *Chen*, *Zuo*, and *Shi* perform their own functions and cooperate with each other (Fan et al., 2006). In summary, the holistic mechanism of SZRP was first studied in a systematic way by network pharmacology.

## Discussion

In this study, the anti-insomnia mechanism of SZRP was first studied in a distinctive way by networking pharmacology. As a prevalent sleeping disorder, insomnia can be induced by various risk factors. Our research found that multiple pathways were involved in the anti-insomnia effects of SZRP. Five herbs in SZRP seem to play different roles in a complimentary way. The ingredients of the main herb mainly targeted multi-neurotransmitter proteins at the pre-synapse and post-synapse interface exerting sedative and hypnotic effects. This effect was likely enhanced by additional herbs through holistically regulating the nervous system related to insomnia symptoms. On top of that, the additional herbs were suggested to intensely regulate the immune system, particularly inflammation cytokines, which were often reported as an important influential factor for insomnia (Quartana et al., 2015; Irwin et al., 2016; Fernandez-Mendoza et al., 2017). Moreover, the whole formula can target the endocrine system to balance the hormone, blood pressure, and blood sugar, enhancing the sedative and hypnotic effects of the main herb.

By decomposing the formula, the *Emperor* (*Jun*) herb, *Z. jujuba*, was found to target synapse membrane, which may fight the main symptom of insomnia. The *Minister* (*Chen*) herbs, *A. asphodeloides* and *W. extensa*, not only can regulate the nervous system helping *Jun* herb in relieving the main symptom but also can densely target inflammation and endocrine pathways treating the likely pathological causes or secondary symptoms. *A. asphodeloides* is reported to act on HRH, which is an effective sedative target (Krystal et al., 2013). Interestingly, only the *Adjuvant* (*Zuo*) herb can uniquely upregulate IL-10, the well-known inflammation-inhibiting factor (Ouyang et al., 2011; Iyer and Cheng, 2012) that seems to counteract the inflammation-inhibiting effects of other herbal groups. It is noted that no unique function has been found for the *Courier* (*Shi*) herb *G. uralensis* from current MOA networking. We suggest that its possible role in ADME processes cannot be detected in the current analysis (Shen et al., 2012; Bi et al., 2014). The most interesting is to see the targeting patterns in the subnetworks among the herbal groups, such as targeting multi-points of the same pathway, parallel pathways, and cross-talked pathways. These patterns have been reported to have beneficial synergistic effects (Jia et al., 2009) and have been applied to design synergistic drug combinations for cancer (Sun et al., 2015).

Meanwhile, the above results seem to support the TCM theory that the *Emperor* (*Jun*) herb often deals with the main symptoms of a disorder, while the *Minister* (*Chen*) assists the *Emperor* (*Jun*) herb to fight the main symptoms and also to remove those accompanying symptoms and signs. The *Adjuvant* (*Zuo*) herbs usually coordinate the effects of the *Emperor* (*Jun*) and *Minister* (*Chen*) herbs by counteracting their toxic or side effects, and the *Courier* (*Shi*) herb helps to deliver or guide the other herbs in the prescription to the target organs (Fan et al., 2006). As such, the future design of anti-insomnia drugs may cover not only the nervous system but also the endocrine and immune systems holistically for better clinical efficacy.

Herbal medicines are usually believed to be multi-functional because of their vast chemical diversity. In this work, we only focused on the widely reported anti-insomnia effects of SZRP. This does not exclude the possibility of additional functions of the formulae. In the pathway enrichment analysis, cancer, cardiovascular disease, signal transformation, and lipid metabolism were also detected for the whole formulae, inferring potential functions to be discovered. In fact, one is often involved with multiple functions and labeled with many different pathways. For instance, NFKB1 was labeled by 67 pathways in KEGG, suggesting its important role as a cross-talk gene. In our case, the ratio of NEI targets chosen for anti-insomnia analysis is about 51.7% of total unique targets.

It is realized that our study aims to explain the global mechanism of traditional formula by decomposing the herbal formula. The current conclusion may be limited by the data quality and quantity, as well as clinical evidence. To avoid noise induction, we started with a widely acknowledged formula as an example, and their targets with literature support, instead of predicting targets. In the future, more efforts should be devoted to mutual interactions between herbal constituents and the dosage effects. Therefore, we here recommend our method of TCM formula with clear clinical effects and validated targets at the current stage.

## Author Contributions

JG wrote the manuscript and contributed to the overall design. QW, YH, and JG analyzed and interpreted the results. YH, KT, and QW modified the manuscript. XY and ZC supervised the project. All authors read, critically reviewed, and approved the final manuscript.

## Funding

This work was supported in part by the National Key R&D Program of China (2017YFC1700200 and 2017YFC0908400) and the National Natural Science Foundation of China (31671379).

## Conflict of Interest Statement

The authors declare that the research was conducted in the absence of any commercial or financial relationships that could be construed as a potential conflict of interest.
